# Pathogenicity of two recent Western Mediterranean West Nile virus isolates in a wild bird species indigenous to Southern Europe: the red-legged partridge

**DOI:** 10.1186/1297-9716-42-11

**Published:** 2011-01-18

**Authors:** Elena Sotelo, Ana Valeria Gutierrez-Guzmán, Javier del Amo, Francisco Llorente, Mehdi El-Harrak, Elisa Pérez-Ramírez, Juan Manuel Blanco, Ursula Höfle, Miguel Angel Jiménez-Clavero

**Affiliations:** 1 Centro de Investigación en Sanidad Animal del Instituto Nacional de Investigación y Tecnología Agraria y Alimentaria (CISA-INIA), Ctra Algete-El Casar, s/n, 28130 Valdeolmos (Madrid), Spain; 2 Instituto de Investigación en Recursos Cinegéticos IREC (CSIC-UCLM-JCCM), Ronda de Toledo s/n, 13005 Ciudad Real, Spain; 3 Biopharma Laboratory, Km 2 Route de Casablanca, BP 4569 Rabat Akkari, Morocco; 4 Centro de Estudios de Rapaces Ibéricas CERI (JCCM), Sevilleja de la Jara, 45671 Sevilleja de la Jara, Spain

## Abstract

West Nile virus (WNV) is an emerging zoonotic pathogen whose geographic spread and incidence in humans, horses and birds has increased significantly in recent years. WNV has long been considered a mild pathogen causing self-limiting outbreaks. This notion has changed as WNV is causing large epidemics with a high impact on human and animal health. This has been particularly noteworthy since its introduction into North America in 1999. There, native bird species have been shown to be highly susceptible to WNV infection and disease with high mortalities. For this reason, the effect of WNV infection in North American bird species has been thoroughly studied by means of experimental inoculations in controlled trials. To a lesser extent, European wild birds have been shown to be affected clinically by WNV infection. Yet experimental studies on European wild bird species are lacking. The red-legged partridge (*Alectoris rufa*) is a gallinaceous bird indigenous to the Iberian Peninsula, widely distributed in South Western Europe. It plays a key role in the Mediterranean ecosystem and constitutes an economically important game species. As such it is raised intensively in outdoor facilities. In this work, red-legged partridges were experimentally infected with two recent WNV isolates from the Western Mediterranean area: Morocco/2003 and Spain/2007. All inoculated birds became viremic and showed clinical disease, with mortality rates of 70% and 30%, respectively. These results show that Western Mediterranean WNV variants can be pathogenic for some European bird species, such as the red-legged partridge.

## Introduction

West Nile virus (WNV) is a neurotropic arthropod-borne virus belonging to the family *Flaviviridae*, genus *Flavivirus*. Its natural cycle involves multiple species of birds as amplifying hosts and a wide range of mosquitoes as vectors. Horses and humans are susceptible to WNV disease but generally are not competent amplifying hosts. In the last fifteen years WNV has expanded its geographic range dramatically, and is now considered the most widespread arbovirus in the world [1-3]. In parallel, significant changes have been observed in its epidemiology, virulence and range of host species affected. While originally WNV was considered a pathogen of little importance, causing mild disease in humans and horses during sporadic, self-limiting outbreaks; this notion is now changing as WNV is causing large epidemics with thousands of human and veterinary cases with associated morbidity and mortality. Large-scale WNV epidemics have taken place since its introduction in North America in 1999, although vaccination of horses has mitigated the effect of this epizooty in this continent. In Europe, where this disease was disregarded for decades, significant changes have occurred, with an apparent increase in the number of outbreaks. Although enhancement of surveillance for this disease in Europe could in part explain this increase, nevertheless, newly observed patterns such as the raising degree of human involvement and an upsurge of pathogenicity for wild birds [4] might mean that the situation with regard to this disease in Europe is in fact changing. In particular, wild bird affection has been observed only recently in a number of cases, caused either by WNV lineages already circulating in Europe [5-8] or by newly introduced lineages [5,9].

One of the hallmarks of WNV epidemiology in North America is that native wild birds are largely affected, in contrast with the low impact of associated disease in wild birds in the Old World [3]. It remains unclear whether this difference is due to the intrinsic pathogenicity of the different viruses found in each continent, or, alternatively, to the differing susceptibility of Palearctic vs. Nearctic bird species to WNV infection. However, while the effects of WNV infection on North American bird species have been intensively studied and described in a number of reports [10-15]), experimental studies on indigenous wild birds are lacking for European birds and WNV strains.

The red-legged partridge (*Alectoris rufa*) is a gallinaceous bird native to the Iberian Peninsula, widely distributed in South Western Europe, and has been introduced to southern England and the Canary Islands [16]. The species plays a key role in Spanish Mediterranean ecosystems and together with the European rabbit (*Oryctolagus cuniculus*) constitutes a staple prey for a number of endangered predators, including the Spanish Imperial eagle (*Aquila adalberti*) and the Iberian lynx (*Lynx pardinus*). Also, it is one of the most important small game species in Central and Southern Spain, where hunting, apart from agriculture, is the most important source of income [17]. Due to the decline of natural populations of this species in most of its distribution range, partridges are raised intensively in outdoor operations and released for hunting purposes and for restocking natural populations [18]. The susceptibility of this species to WNV infection is unknown. However, a recent study reported an outbreak of clinical disease in North America caused by WNV infection in Chukar partridges (*Alectoris chukar*) [19], a species closely related to the red-legged partridge. These data prompted us to investigate the effects of WNV infection in European red-legged partridges. For that purpose we selected two Western Mediterranean strains of WNV: Morocco/2003 and Spain/2007, isolated in the region corresponding to the natural geographic range of red-legged partridges. Both strains are highly related phylogenetically [20]. The present study describes the course of an experimental infection of red-legged partridges with the two Western Mediterranean West Nile virus strains selected. The occurrence of direct contact transmission was also assessed.

## Materials and methods

### Viruses and virus preparations

The viruses used in this study were WNV Spain/2007 (strain GE-1b/B, GenBank acc. n°: FJ766331) [20] and WNV Morocco/2003 (strain 04.05, GenBank acc. n°: AY701413) [21]. These two strains are highly related phylogenetically, differing in 13 amino acid positions throughout the complete genome (3433 amino acid residues), one of them located at the NS3_249_. This site has been involved in genetic modulation of WNV pathogenicity, using American crows (*Corvus brachyrhynchos*) as avian model of WNV disease [22]. For preparation of inocula, WNV Spain/2007 was cultivated in BSR cells (clone of BHK-21 cell line) and WNV strain Morocco/2003 was cultivated in Vero cells. Both strains were titrated by plaque assays in Vero cells as previously described [23]. Virus titers are given in pfu (plaque-forming units) per mL. WNV Spain/2007 and Morocco/2003 strains of low passage number (3 and 5 cell culture passages, respectively) were used for inoculations. Complete nucleotide sequences of both viruses were analyzed prior to inoculation to ascertain that the viruses inoculated had nucleotide sequences identical to those available in GenBank.

### Experimental inoculations in red-legged partridges

Recently hatched red-legged partridge chicks (n = 36) were obtained from a commercial breeder and raised in the experimental farm of the Instituto de Investigación en Recursos Cinegéticos (IREC) until they were six weeks of age. Prior to the experiment, all individuals were tested serologically (using a commercially available competition ELISA as described below) and virologically (by real-time RT-PCR as described below) to ensure that exposure to WNV had not occurred. The partridges were transported to the biosafety level 3 (BSL-3) facilities in the CISA and randomly assigned to experimental groups, that were housed in wire mesh cages with absorbent floor that was changed daily. The birds were provided with a commercial diet for game birds and water *ad libitum *throughout the experiment. After five days for acclimatization, two groups composed of 10 red-legged partridges each were inoculated subcutaneously (10^4 ^pfu/individual) with either WNV Spain/2007 or WNV Morocco/2003 diluted in up to 0.1 mL in Dulbecco's Minimum Essential Medium (DMEM) (supplemented with 2 mM L-glutamine, 100 U/mL penicillin and 100 μg/mL streptomycin). All partridges were in their seventh week of age when they were inoculated. Both groups were kept in separate cages (120 × 40 × 40 cm). Two groups of three non-inoculated partridges (contact groups) were kept in the same cages as inoculated individuals. One additional group of partridges (n = 10) was sham-inoculated with an equivalent volume of DMEM and kept in a separate cage, as an environmental control. The experiment was performed in the CISA BSL-3 animal facilities, following biosafety, animal welfare and ethical rules applicable in the EU.

### Clinical follow-up and collection of samples

Disease symptoms were observed daily for up to 10 days post-inoculation (dpi). To follow the viremia course, blood samples were collected daily during the same period from the cutaneous ulnar vein at 1, 3, 5, 7 and 9 dpi in half of the individuals, and at days 2, 4, 6, 8 and 10 dpi in the other half (that is, alternating individuals in order to reduce animal manipulation and thus reduce unnecessary stress). A second blood sample (0.1-0.2 mL/individual) was taken likewise in dry tubes and allowed to clot at 37°C for 1 h, followed by incubation at 4°C overnight, in order to obtain serum for antibody detection techniques (ELISA and/or virus-neutralization tests). Similarly, oropharyngeal and cloacal swabs were taken 1 day before inoculation (i.e., -1) from all individuals, and at 4, 6, 10 and 14 dpi in half of the individuals of each group of inoculated partridges, and 3, 5, 7 and 14 dpi in the other half. Blood samples were collected directly in sterile polypropylene tubes filled with 0.9 mL BA-1 diluent (Hanks M-199 salts, 0.05 M Tris, pH 7.6, 1% bovine serum albumin, 0.35 g/L of sodium bicarbonate, 100 units/mL of penicillin, 100 μg/mL of streptomycin, 1 μg/mL of amphotericin B) and stored at -70°C until analysis. Swab samples were placed in sterile polypropylene tubes filled with 1 mL of Hank's balanced solution containing 10% glycerol, 200 U/mL penicillin, 200 μg/mL strepctomycin, 100 U/mL polymixin B sulphate, 250 μg/mL gentamicin and 50 U/mL nystatin and also stored at -70°C until analysis. Contact partridges were sampled (both for blood and cloacal/oropharyngeal swab) at days -1, 6, and 10 dpi. Following death, partridges were collected and necropsied within < 18 h. Two individuals of each contact group were euthanized humanely by intravenous injection of embutramide (T61 ^®^, Intervet - Schering-Plough, Madrid, Spain) on day 10 dpi and submitted for full necropsy. During necropsy, care was taken to avoid cross-contamination of tissues by use of single use scalpels and forceps. After the 10 day period, additional serum samples for the detection of specific antibodies against WNV in surviving partridges were collected on 12, 14, and 20 dpi, in inoculation, contact and control groups.

### Viremia and WNV genome detection assays

Viremia was measured in blood (collected in BA-1 diluent) by standard plaque-formation assays as described [23] while viral genome load was measured in blood (collected in BA-1 diluent) by semiquantitative real-time RT-PCR, using a previously described method [24] with slight modifications, using an internal control. Briefly, nucleic acids were extracted from BA-1-diluted blood samples (0.1 mL) using Biosprint-15 automated extraction (QIAGEN, Valencia, CA, USA), and 4 μL (4% of total eluate) of each extract, in 25 μL final volume, was subjected to real-time RT-PCR amplification using the QuantiTect Probe RT-PCR kit (QIAGEN) and following manufacturer's instructions. The mix included primers (WNV-LCV-F1: 5'-GTGATCCATGTAAGCCCTCAGAA-3' and WNV-LCV-R1: 5'-GTCTGACATTGGGCTTTGAAGTTA-3') at 0.4 μM final concentration, and a FAM-labelled TaqMan-MGB probe (WN-LCV-S1: 5'FAM-AGGACCCCACATGTT-3'-MGB) at 0.25 μM final concentration, specific for detection of lineage 1 WNV, as well as primers (ACT F 1005-1029: 5'-CAGCACAATGAAGATCAAGATCATC-3', and ACT R 1135-1114: 5'-CG GACTCATCGTACTCCTG CTT-3') and JOE-labelled TaqMan probe (ACT P 1081-1105: 5'JOE-TCGCTGTCCACCTTCCAGCAGATGT-BBQ) for detection of an internal control (β-actin) as described [25] in order to control for the presence and integrity of RNA isolated from each preparation. Amplification conditions consisted of a first reverse-transcription step at 50°C for 30 min, followed by 15 min at 95°C ("hot start") and 45 cycles of 15 s at 95°C and 1 min at 60°C. Tissues, oropharyngeal and cloacal swabs were examined for the presence of viral genome using the same RNA extraction method and real-time RT-PCR described above, but following a preparation step consisting of homogenization for 2 min at 30 cycles/s in 90% phosphate-buffered saline (PBS) using a Tissuelyser homogenizer, QIAGEN, followed by clarification at 850 × *g *10 min. in the case of tissues, and elution for 3 h at 4°C in 1 mL BA-1 diluent (see above), followed by clarification at 9500 × *g *5 min, in the case of oropharyngeal and cloacal swabs.

### Antibody detection assays

Serum antibodies to WNV were detected by a commercially available competition ELISA suitable for measurement of antibodies to WNV in serum of wild birds following manufacturer's instructions (ID Screen^© ^West Nile Competition, IdVet, Montpellier, France). Virus-neutralizing antibody titers were measured by a micro-virus neutralization test as described [26]. Positive neutralization was considered for each inoculated well when complete absence of CPE was observed in the cell monolayer.

### Statistical analysis

Comparison of survival rates between groups of partridges inoculated with Morocco/2003 and Spain/2007 WNV strains was carried out by using the Gehan-Breslow-Wilcoxon method [27]. Viremia and genome load data between groups of partridges inoculated with Morocco/2003 and Spain/2007 WNV strains were compared using Student's *t *test for independent groups.

## Results

**Figure 1:**
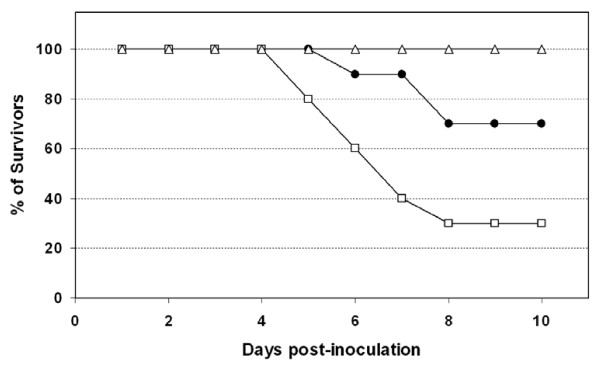
**Survival of red-legged partridges as a function of infection with either West Nile virus (WNV) isolate Morocco/2003 (open squares) or WNV isolate Spain/2007 (closed circles)**. Open triangles represent sham-inoculated partridges acting as non-infected controls. The percentage of surviving red-legged partridges in each group is plotted against day post-infection.

**Figure 2:**
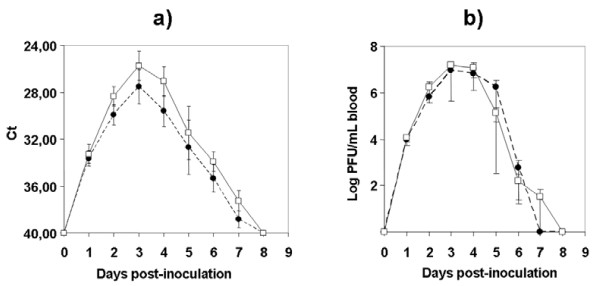
**Mean daily blood viral genome load (a) and viremia titers (b), plotted for two groups of 10 red-legged partridges: one inoculated with WNV Morocco/2003 (open squares) and another inoculated with WNV Spain/2007 (closed circles)**. Each group was alternatively sampled every other day as described in the text. Each point represents the mean of five individuals, or of the surviving individuals of each group. Error bars represent the standard error of the mean. The efficiency of the semi-quantitative real-time RT-PCR assay for viral genome load determinations was E = 0.908.

**Figure 3:**
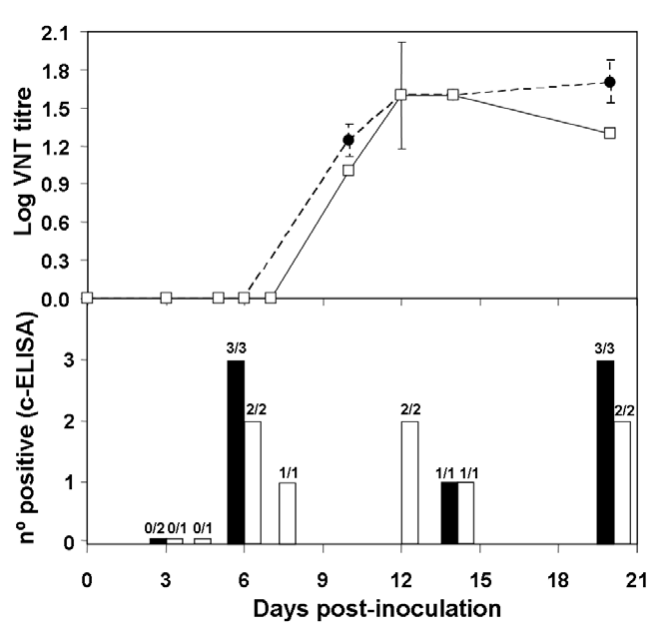
**Course of the antibody response to WNV in serum from inoculated red-legged partridges, as measured by virus-neutralization test (VNT, expressed as log titres) and competitive ELISA (cELISA, expressed as n° of positives)**. Upper panel: Solid line/open squares represent the neutralizing antibody response in individuals inoculated with WNV Morocco/2003 strain; dashed line/closed circles represent the neutralizing antibody response in individuals inoculated with WNV Spain/2007 strain (error bars: standard deviation, only available for those data where n ≥ 3; n: 1-5). Lower panel: ELISA results are represented by black bars (sera from individuals inoculated with WNV Spain/2007 strain) and white bars (sera from individuals inoculated with WNV Morocco/2003 strain). Over the bars, numbers of positive/examined serum samples are indicated.

**Figure 4:**
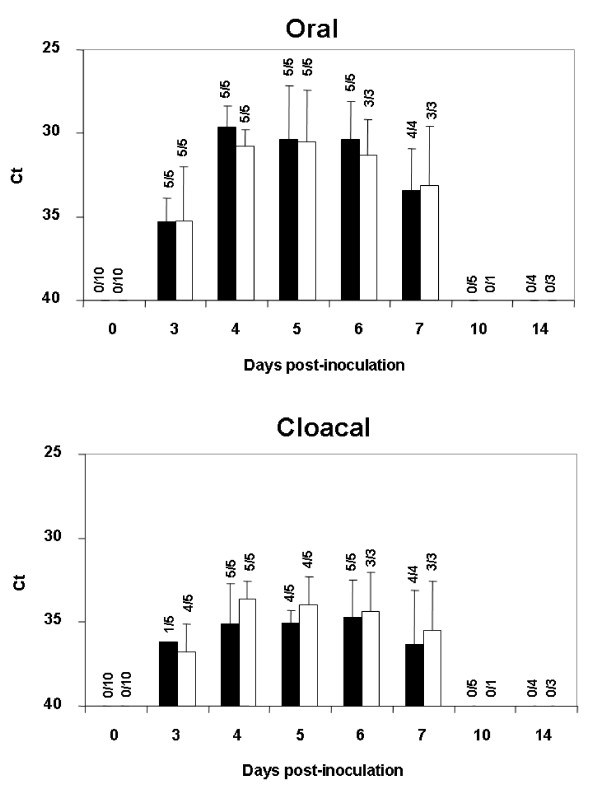
**Viral shedding through the oral (upper panel) and fecal (lower panel) routes, as estimated by real-time RT-PCR analysis of the oropharyngeal and cloacal swabs, respectively, taken at different days post-inoculation of WNV Spain/2007 strain (black bars) or WNV Morocco/2003 strain (white bars)**. Error bars represent standard deviation of the means. Over the bars, numbers of positive/examined swab samples are indicated.

Both WNV strains assayed were pathogenic for red-legged partridges. All inoculated partridges showed clinical signs (e.g. loss of appetite, ruffled feathers, paralysis, and unresponsiveness), yet those infected with Morocco/2003 were more severely affected than those infected with Spain/2007. Firstly, the onset of symptoms began one day earlier (4 vs. 5 dpi) in partridges inoculated with Morocco/2003 than in those inoculated with Spain/2007. Secondly, mortality was higher (70% vs. 30%) and started earlier (5 vs. 6 dpi) with Morocco/2003 than with Spain/2007 (Figure 1). The differences observed in survival rates over time between both groups of inoculated partridges were statistically significant (p < 0.05). Clinical signs and mortality ceased by 9 dpi in both inoculated groups. Viral genome was detectable from 1 to 7 dpi (Figure 2a) in both inoculations. Viremia was detectable between 1 and 6 dpi for Spain/2007 and slightly longer (up to 7 dpi) for Morocco/2003. Peak viremia titers, and peak viral genome loads were found at 3 dpi for both isolates. Mean peak viremia was higher after inoculation with Morocco/2003 (10^7.2 ^pfu/mL) than with Spain/2007 (10^6.9 ^pfu/mL) (Figure 2b), similarly to what occurred with viral genome loads (Figure 2a) although in both cases the differences between both groups were not statistically significant (p > 0.05). All surviving partridges developed WNV-neutralizing antibodies (NtAb) (Figure 3), which were observed at 10 dpi or later. In inoculated partridges, specific antibodies to WNV as revealed by competitive ELISA were observed slightly earlier (6 dpi) than NtAb (Figure 3). Oral and cloacal virus shedding was observed between 3 and 7 dpi for both viruses. Virus shedding was more consistently found in oropharyngeal than in cloacal swabs for both strains, particularly in early stages (3 dpi, Figure 4). *Post-mortem *examination of lethally infected partridges revealed systemic infection, as the virus was detected in the brain, heart, spleen and liver by means of real time RT-PCR (Table 1). Upon necropsy, infected partridges were in poor body condition. Gross lesions were similar in both WNV-inoculated groups, and were characterized mainly by ascites, diffuse pallor of the myocardium, enlarged liver and petechia on the renal surface, injected blood vessels in the intestinal tract, and, in three birds, congestion of superficial cerebral vessels.

**Table 1:** Summary of the WNV genome detection by real-time RT-PCR performed on tissues from inoculated red-legged partridges.

		Results (**)			
		
Individual identification	Inoculum	Brain	Heart	Kidney	Spleen
					
10	Spain/2007(*)	Pos	Pos	Pos	Pos
11	Spain/2007(*)	Pos	Pos	Pos	Pos
13	Spain/2007(*)	Pos	Pos	Pos	Pos
20	Morocco/2003 (*)	Pos	Pos	Pos	Pos
24	Morocco/2003 (*)	Pos	Pos	Pos	Pos
29	Morocco/2003 (*)	Pos	Pos	Pos	Pos

* Contact (non-inoculated cage-mate) controls gave negative results upon necropsy at 10 dpi in all tissues analyzed by real-time RT-PCR.

**Samples were considered positive ("Pos") at Ct ≥ 40.0.

The virus was not detectable in blood of non-inoculated contact partridges during the whole course of the experiment, and, furthermore, these partridges did not seroconvert, as assessed either by competition ELISA or by virus-neutralization test (data not shown), further supporting the absence of infection. Oropharyngeal and cloacal swabs from these contact partridges also remained negative until they were euthanized for tissue examination. Brain, heart, spleen and liver samples examined from these birds resulted negative for WNV genome by real-time RT-PCR at 10 dpi (Table 1). Sham-inoculated partridges (environmental control group) did not show symptoms or mortality, and remained negative for virus genome and antibody detection throughout the experiment.

## Discussion

The results of this work demonstrated that the red-legged partridge is susceptible to WNV disease, and provides further evidence that some European wild bird species can be clinically affected by WNV. This concept is supported by recent reports describing field WNV cases in different wild bird species in Europe. While previous work reported three types of European eagles (golden eagle -*Aquila chrysaetos*-, Spanish imperial eagle -*Aquila adalberti*- and Bonnelli's eagle -*Hieraaetus fasciatus-*) clinically affected by WNV in Spain [6,7], the disease was also described in goshawks (*Accipiter gentilis*), sparrow hawks (*Accipiter nisus*) and geese (*Anser anser*) in Hungary [5], sparrow hawks in Austria [9], and house sparrows (*Passer domesticus*) and magpies (*Pica pica*) in the Camargue region of France [8]. Just prior to the arrival of WNV to North America in 1999, migrating storks and domestic geese were adversely affected by WNV infection in Israel [28]. Subsequent experimental studies on domestic geese conducted in Israel confirmed their susceptibility to WNV disease [29]. Nevertheless, to our knowledge, the present study is the first reporting an experimental infection with WNV in a European wild bird showing symptoms of disease.

In addition, our results demonstrate experimentally that at least some Euro-Mediterranean WNV strains are pathogenic in an indigenous wild bird species. Previous results showed that one of the strains used in this experiment (Morocco/2003) was as pathogenic for mice as the NY99 prototype strain [20]. Thus, the apparently limited wild bird morbidity caused by WNV in Europe, as compared to the high virulence for WNV in wild birds in North America, demands an explanation accounting for the fact, reflected in this work, that at least some Euro-Mediterranean WNV strains are highly pathogenic for certain indigenous wild birds such as the red-legged partridge. In the European context the apparently mild consequences of WNV disease for wild birds would likely arise from the difficulties inherent to the surveillance of mortality events in the affected wild bird species rather than from low pathogenicity of local WNV strains, or from low susceptibility of indigenous wild birds.

In this work, contact partridges did not show disease symptoms, and remained negative both for virus detection in blood and/or tissues, as well as for WNV-specific antibodies. Thus, direct WNV transmission did not occur in the experimental conditions used in this assay, despite close interaction between inoculated and non-inoculated partridges. While contact transmission has been demonstrated for certain avian species [29], evidence of this is lacking for other wild bird species. For instance, in an exhaustive study evaluating WNV transmission dynamics in 25 different avian species, contact (cage-mate) transmission was observed in only four species (Blue Jays, Black-billed Magpies, American Crows and Ring-billed Gulls). Interestingly, contact transmission was not observed in the two species of Galliformes evaluated (Japanese Quail and Northern Bobwhite) [10]. In a different study addressing long-term immunity in House Sparrows as important amplifying hosts for WNV, contact transmission was not observed in this species [14]. Viral shedding by the oral and faecal routes occurred in all the inoculated partridges monitored, so the absence of contact transmission in this model needs further investigation, in particular if this finding is attributable to the avian model, or to the viral isolates used.

New animal models of WNV infection are of interest not only for virulence studies but also for antiviral and vaccine development [30]. This work demonstrates that the red-legged partridge constitutes a suitable avian model of WNV infection, since this bird species is not only susceptible to infection and disease but is also easy to obtain, can be raised in captivity, and is relatively easy to handle. In addition, our data demonstrate that the red-legged partridge could act as amplifying host for WNV, as shown by the maximum viremia levels attained during the experiment (approximately 10^7.0 ^pfu/mL blood), clearly above the established threshold level of infectious viremia, estimated at 10^5.0 ^pfu/mL of blood [10].

The red-legged partridge is indigenous to the Mediterranean region, where it constitutes a key prey species for many predators. Those predators feeding on infected partridges or carrion eaters feeding on their carcasses could be at risk of infection since non-vectored transmission of WNV through ingested infected food has been documented [10,31,32]. In Spain, some endangered species such as the Golden eagle, the Bonnelli's eagle and the Spanish Imperial eagle prey regularly on red-legged partridges and have been shown to be susceptible to WNV-associated disease [6,7]. Thus the occurrence of WNV outbreaks in red-legged partridges could potentially constitute a risk for the conservation of these endangered species.

The red-legged partridge also represents an important small game species being intensively raised in open air facilities and released into the wild for restocking and hunting purposes. The species also exists in the South of the UK and the Canary Islands, where there is concern about the introduction and spread of WNV. A closely related partridge species (Chukar partridge) is also susceptible to WNV-associated disease in North America, where farms raising these wild birds have suffered outbreaks of the disease [19]. Besides their economic importance, partridge farms could play a relevant role for public health since in case of a hypothetical WNV outbreak, and provided the presence of suitable mosquito species with a broad host spectrum, farmed red-legged partridges could potentially act as the reservoir for accidental transmission of WNV into humans and horses. Suitable mosquito vectors as *Culex pipiens *are abundant in the red-legged partridges' natural habitat as well as in the farms (unpublished observation). Thus, in surveillance programs, WNV disease should be considered as a potential hazard for farms producing red-legged partridges. This is of particular importance bearing in mind that eventually these captive-raised partridges are to be released into the wild.

In the experimental work described here, the viral strains (Morocco/2003, and Spain/2007) used to inoculate differed in 13 out of 3433 amino acid residues (99.62% amino acid identity), one of them being NS3_249_. This site appears to play a role in the genetic modulation of pathogenicity for wild birds, as substitution of threonine (T) by proline (P) in a moderately pathogenic WNV strain (Ken98) resulted in an increase in virulence for American crows, whereas, the opposite change (P249T) resulted in a decrease in virulence for the highly pathogenic NY99 strain in the same hosts [22]. This site is occupied by T in Morocco/2003, and by P in Spain/2007. We showed recently in a mouse model that Morocco/2003 is more pathogenic than Spain/2007 [20]. Here we confirm and extend this observation to a wild bird species. This suggests that a proline residue in position 249 of the NS3 protein is not sufficient to enhance virulence for any given WNV strain. Possibly, other residues that differ between both strains are also playing a role in the observed pathogenicity, and could have produced apparent discrepancies with previous studies using infectious clones in American crows [22,33]. The NS3_249_-P genotype has arisen recently in the Western Mediterranean region. This WNV genotype has been involved in recent outbreaks in northeastern Italy [34], in which WNV strains have been similar to those used in the present study [20]. An assessment of the pathogenicity of these recent Italian isolates would provide further insight into the epidemiology of the NS3_249 _genotype in the Western Mediterranean area.

The pathogenicity of the Spanish WNV isolate was less than that of another Western Mediterranean strain, i.e., Morocco/2003, not only in a mouse model, but also in the red-legged partridge. This finding could explain why this disease has remained essentially silent in Spain to date, and provide a possible mechanism accounting for the occurrence of sporadic WNV outbreaks interspersed by silent WNV circulation in some regions such as the Western Mediterranean. Small genetic changes in the virus genome (T249P at NS3, or others), could substantially reduce the pathogenicity of circulating viruses, and vice-versa. However, other factors not linked to the virus, but to host susceptibility and vectors, could also contribute to this epidemiological pattern, the relative importance of which merits further investigations.

## Competing interests

The authors declare that they have no competing interests.

## Authors' contributions

ES prepared the inocula, carried out animal experimental work, and performed most virological, molecular and serological analyses. AVG performed animal experimental work and participated in the pathological examination of post-mortem specimens. JD performed animal experimental work and participated in a part of the molecular analyses. FL performed animal experimental work, carried out part of the serological tests and performed the statistical analysis. ME prepared one of the viral stocks used in the inoculations and helped in the design of the study. EP participated in the animal experimental work. JMB raised the red-legged partridges. UH participated in the design and coordination of the study, red-legged partridge raising and pathological examination of post-mortem specimens. MAJ conceived of the study, participated in its design and coordination, and drafted the manuscript. All authors read and approved the final manuscript.
